# Trends, characteristics, in-hospital outcomes and mortality in surgical mitral valve replacement among patients with and without COPD in Spain (2001-2015)

**DOI:** 10.1371/journal.pone.0221263

**Published:** 2019-08-19

**Authors:** Javier de-Miguel-Díez, Ana López-de-Andrés, Valentín Hernández-Barrera, José M. De Miguel-Yanes, Manuel Méndez-Bailón, Nuria Muñoz-Rivas, Rodrigo Jiménez-García

**Affiliations:** 1 Respiratory Department, Hospital General Universitario Gregorio Marañón, Facultad de Medicina, Universidad Complutense de Madrid (UCM), Instituto de Investigación Sanitaria Gregorio Marañón (IiSGM), Madrid, Spain; 2 Preventive Medicine and Public Health Teaching and Research Unit, Health Sciences Faculty, Rey Juan Carlos University, Alcorcón, Madrid, Spain; 3 Internal Medicine Department, Hospital General Universitario Gregorio Marañón, Madrid, Facultad de Medicina, Universidad Complutense de Madrid (UCM), Spain; 4 Internal Medicine Department, Hospital Universitario Clínico San Carlos, Facultad de Medicina, Universidad Complutense de Madrid (UCM), Madrid, Spain; 5 Medicine Department, Hospital Universitario Infanta Leonor, Madrid, Spain; Oregon Health & Science University, UNITED STATES

## Abstract

**Purpose:**

We examined trends, characteristics and in-hospital outcomes in mechanical and bioprosthetic surgical mitral valve replacement (SMVR) among patients with and without chronic obstructive pulmonary disease (COPD) in Spain from 2001 to 2015. We also identified factors associated with in-hospital mortality (IHM) in both groups of patients according to the implanted valve type.

**Methods:**

We analyzed data from the Spanish National Hospital Discharge Database for patients aged 40 years or over. We selected admissions of patients whose medical procedures included SMVR. We grouped hospitalizations by COPD status.

**Results:**

Over 43,024 patients identified, 83.63% underwent mechanical mitral valve replacement and 16.37% bioprosthetic valve (6.71% and 7.78% with COPD, respectively). The incidence of SMVR decreased for mechanical valves and increased for bioprosthetic valves over time in both groups of patients. The incidence of SMVR admissions was lower among COPD patients than in those without COPD, both for mechanical and bioprosthetic valves. IHM decreased significantly over time, regardless of the type of valve, in both groups of patients. COPD was associated with a significant increase in IHM, but only among patients who underwent bioprosthetic SMVR (OR 1.32, 95% CI 1.01–1.73).

**Conclusions:**

The incidence of mechanical SMVR decreased while that of bioprosthetic SMVR increased over time in both groups of patients. COPD patients were less surgically operated than non-COPD patients for both valve types. In COPD patients, bioprosthetic SMVR was proportionally more used than mechanical SMVR. Mortality decreased over time for both valve types in patients with and without COPD. COPD increased in-hospital mortality among patients undergoing a biological SMVR.

## Introduction

Mitral valve disorder is one of the most common heart valve diseases in the developed countries [1]. About 2% of the general population has significant mitral valve disease [2]. Incidence increases in the elderly, affecting nearly 10% of people above the age of 75 years [3]. Surgery remains the gold standard treatment [4]. Valve replacement prolongs survival among patients with severe involvement [5]. Valve prostheses can be mechanical or biologic, and each one has risks and benefits. Bioprosthetic valves are subjected to structural degeneration and may require future reoperation, while mechanical valves typically require lifelong anticoagulation with associated risks of thromboembolic and bleeding [5,6]. Despite it, the use of biologic prostheses has increased substantially for surgical mitral valve replacement (SMVR) in recent years [5,7].

Among the factors associated with poor SMVR outcomes are advanced valvular cardiomyopathy, age, and associated comorbidities [6]. In fact, a substantial part of symptomatic patients with severe mitral valve disease are excluded from surgery by institutional heart teams for these reasons [8,9]. Chronic obstructive pulmonary disease (COPD) is one of the comorbidities that has been associated with the decision to not operate [9]. However, in a recent study, Mkalaluh et al [10] analyzed a contemporary series of octogenarians undergoing mitral valve surgery at their institution and found that COPD did not influence 30-day mortality.

Understanding the changing characteristics and the prognosis of these patients is important to interpret previous studies and design future trials [11]. However, nationwide data on trends in SMVR are scarce [12]. The aim of the present study was to examine nationwide trends, characteristics and in-hospital outcomes in mechanical and bioprosthetic SMVR among patients with or without COPD from 2001 to 2015 using the Spanish National Hospital Discharge Database (SNHDD). Also we identified factors associated with in-hospital mortality (IHM) among patients with and without COPD according to the implanted valve type.

## Material and methods

### Data source

The SNHDD covers over 95% of hospital admissions in Spain and contains nationwide information of up to 14 discharge diagnoses and up to 20 procedures performed during the hospital stay including admission and discharge dates [13]. International Classification of Diseases, Ninth Revision, Clinical Modification (ICD-9-CM) is used for coding in the SNHDD. For this retrospective study, we used data from years 2001 through 2015.

### Study population

We selected admissions of patients (aged ≥40 years) whose medical procedures included mitral valve replacement (ICD-9-CM codes: 35.23 and 35.24) in the SNHDD database.

We grouped admissions by COPD status as follows: COPD sufferers those patients with any of the following ICD-9-CM codes in any diagnostic position: 490, 491, 491.0, 491.1, 491.2x, 491.8, 491.9, 492, 492.0, 492.8 and 496 or non-COPD those without these codes.

### Covariates

Clinical characteristics included information on overall comorbidity at the time of discharge, which was assessed by calculating the Charlson comorbidity index (CCI) [14]. Calculation of the CCI was done excluding COPD as defined before as a disease.

Among the procedures we specifically analysed if Coronary Artery Bypass Graft (CABG), Surgical Aortic Valve Replacement (SAVR), surgical procedures on pulmonary and/or tricuspid valves, intra-aortic balloon counter-pulsation, pacemaker implantation and blood transfusions were done during the hospitalization for SMVR.

ICD-9-CM codes for conditions included in the CCI as well as other diseases and procedures performed during the hospital stay and analyzed in this investigation are shown in Table 1.

**Table 1:** Diagnosis and procedures with corresponding ICD-9-CM codes.

	ICD-9-CM codes
Chronic obstructive pulmonary disease	490, 491, 491.0, 491.1, 491.2x, 491.8, 491.9, 492, 492.0, 492.8, 496
Type 2 diabetes mellitus	250.x0 and 250.x2
Peripheral vascular disease	0.93.0,473.3,440.x,441.x,443.1–443.9,447.1,557.1,557.9,V43.4
Renal disease	403.01, 403.11, 403.91, 404.02, 404.03, 404.12, 404.13, 404.92, 404.93, 582, 583.0–583.7, 585, 586, 588.0, V42.0, V45.1, V56
Cerebrovascular disease	362.34, 430.x–438.x
Congestive heart failure	398.91,402.01,402.11,402.91,404.01, 404.03,404.11,404.13,404.91,404.93,425.4–425.9, 428.x
Ischemic stroke	433.xx, 434,xx, 436
Atrial fibrillation	427.31
Pulmonary hypertension	416.0 and 416.8
Coronary artery disease	410.xx, 412.x, 413.x, 414.0, 414, 414.00, 414.01, 414.2–9
Acute myocardial infarction	410.xx
Obesity	278.0
Cardiogenic shock	785.51
Gastrointestinal hemorrhage	578, 578.0, 578.1, 578.9
Endocarditis	424.90, 421.9, 424.1, 421.0
Pneumonia	480–488, 507.0–507.8
Acute renal disease	584, 584.5–584.9
Liver disease	070.22, 070.23, 070.32, 070.33, 070.44, 070.54, 070.6, 070.9, 456.0–456.2, 570.x, 571.x, 572.2–572.8, 573.3, 573.4, 573.8, 573.9, V42.7
Cancer	140.x–172.x, 174.x–195.x, 196.x–199.x
Weight loss	260, 261, 262, 263.0–263.2, 263.8, 263.9, 783.2, 977.4
Coronary artery bypass graft	36.10–36.19
Surgical aortic valve replacement	35.21, 35.22
Other valves procedures on pulmonary or tricuspid valves	35.33, 35.03, 35.04, 35.13, 35.14, 35.25, 35.26, 35.27, 35.28, 35.33
Intra-aortic balloon counterpulsation	37.61
Pacemaker implantation	37.70–37.74; 37.80–37.83
Blood transfusion	99.00, 99.01–99.08

We evaluated the mean of length of hospital stay (LOHS) and we estimated the incidence of peri-operative Major Adverse Cardiovascular and Cerebrovascular Events (MACCE) as described by Newman et al [15], MACCE includes acute myocardial infarction, ischemic stroke or death during the hospitalization.

### End points

The main end points in our investigation were trends, characteristics, in-hospital outcomes, and IHM in SMVR among patients with and without COPD. IHM was defined by the proportion of patients who died during admission for each year of study.

### Statistical analysis

The analyses were stratified according to the type valve use (mechanical and bioprosthetic) in the valve replacement and for each group of patients (COPD and non-COPD patients). We considered three time-periods that included five consecutive years each (2001–05; 2006–10; 2011–15).

In order to assess time trends we estimated the incidence rates of admission for SMVR in COPD patients and non-COPD patients per 100,000 inhabitants applying the methods described in previous studies and included Poisson regression analysis adjusted by age and sex [16].

A descriptive statistical analysis was performed for all continuous variables and categories. Variables are expressed as proportions as means with standard deviations. A bivariable analysis according to year was performed using the χ^2^ test for linear trend and ANOVA, as appropriate.

To describe the changes observed from period 2001/5 to period 2011/15 we estimated the “Relative Change Overtime (%)” as the difference from period 2011–15 to 2001–05 divided by the figure in period 2011/15. This represents the increase (positive %) or decrease (negative %) for the figures observed in the 2011/15 period with respect to the figures observed in the 2001/5 period.

To identify predictors of IHM we performed multivariable logistic regression analyses, one for each type of mitral valve replacement (mechanical and bioprosthetic) and for each group of patients (COPD patients, non-COPD patients and both). The variables included in the multivariable models were those with significant results in the bivariable analysis and those considered relevant in other investigations. Estimates were Odds Ratios (ORs) with their 95%CI.

### Sensitivity analysis

To avoid the confounding effect of clinical characteristics on our main study outcome variables (MACCE and IHM) we conducted a sensitivity analysis. To do so we used a propensity score matching (PSM) to select a matched non-COPD patient for each COPD patient. PSM was done using multivariable logistic regression for mechanical and bioprosthetic SMVR patients separately. The variables included to estimate the PSM were year of surgery, sociodemographic and clinical characteristics shown in Table 1.

For data management we used Access. All statistical analyses were performed with Stata version 10.1 (Stata, College Station, Texas, USA). Statistical significance was set at p<0.05 (2-tailed).

### Ethical aspects

The study maintains data confidentiality at all times. Given the anonymous and mandatory nature of the database, it was not necessary to obtain informed consent or approval by an ethics committee in accordance with Spanish legislation.

## Results

In our study we identified a total of 43,024 hospitalizations of patients aged 40 years or more who underwent SMVR in Spain (2001–2015) of them 2,965 suffered COPD (6.89%).

We identified 35,983 (83.63%) hospitalized patients who underwent mechanical mitral valve replacement (6.71% with COPD) and 7,041 (16.37%) who underwent bioprosthetic mitral valve replacement (7.78% with COPD).

### Trends in mechanical mitral valve replacement hospitalizations

The proportion of patients receiving a mechanical valve decreased from 90.22% in 2001–05 to 77.61% in 2011–15 (p<0.001). Equivalent figures for COPD patients were 88.59% to 74.18% and for non-COPD patients from 90.09% to 77.86% (p<0.001, in both cases).

The incidence of mechanical mitral valve replacement among COPD patients decreased from 11.95 cases per 100,000 individuals in the COPD population in 2001 to 11.56 in 2015. Among, non-COPD patients, the incidence also decreased significantly from 12.77 in 2001 to 9.87 in 2015 (p<0.001) (S1 Fig).

The Poisson regression models yielded an adjusted IRR for patients with COPD who underwent mechanical valve replacement of 0.76 (95%CI 0.73–0.80). Therefore, compared to patients without COPD the incidence of mechanical mitral valve replacement hospitalizations was lower among COPD patients.

In patients who underwent mechanical mitral valve replacement, there was a significant male predominance in COPD patients (67.4%), however in patients without COPD there was female predominance (63.85%). Overall, patients with COPD were older (66.5; SD = 8.43 years) than patients without COPD (64.49; SD = 9.43 years; p<0.05) and were more likely to undergo simultaneous CABG (16.26% vs. 10.78%). However, patients without COPD were more likely to undergo other valves procedures on pulmonary or tricuspid valves (25.97% vs. 20.94%) (Table 2).

**Table 2:** Incidence, sociodemographic and clinical characteristics of patients hospitalized that underwent a mechanical mitral valve replacement in Spain from 2001 to 2015 according to COPD status.

	COPD	TOTAL	Relative change over time (%)	Trend
Number of procedures (Incidence per 100,000 inhabitants)*	Yes	2417(8.48)	-47.05	<0.001
	No	33566 (11.12)	-29.02	<0.001
	Both	35983(10.89)	-30.24	<0.001
Age, mean (SD)*	Yes	66.5(8.43)	-0.84	0.003
	No	64.49(9.43)	-1.18	<0.001
Female sex, n (%)*	Yes	788(32.6)	12.32	0.183
	No	21432(63.85)	-6.11	<0.001
Coronary artery bypass graft, n(%)*	Yes	393(16.26)	7.53	0.723
	No	3618(10.78)	10.17	0.012
Surgical aortic valve replacement, n(%)	Yes	778(32.19)	17.87	0.022
	No	10857(32.35)	9.82	<0.001
Other valves procedures on pulmonary or tricuspid valves, n(%)*	Yes	506(20.94)	36.3	<0.001
	No	8718(25.97)	24.96	<0.001
Intra-aortic balloon counterpulsation, n(%)	Yes	82(3.39)	-11.18	0.925
	No	1191(3.55)	13.14	0.081
Pacemaker implantation, n(%)	Yes	114(4.72)	42.16	0.044
	No	1422(4.24)	43.96	<0.001
Blood transfusion, n(%)	Yes	548(22.67)	1.19	0.967
	No	8099(24.13)	4.53	<0.001
Length of hospital stay, mean (SD)*	Yes	23.05(19.72)	-17.86	<0.001
	No	22.02(20.65)	-1.65	<0.001
In-hospital mortality, n(%)	Yes	279(11.54)	-55.88	<0.001
	No	3634(10.83)	-23.03	<0.001
MACCE, n(%)	Yes	368(15.23)	-53.51	0.001
	No	4785(14.26)	-13.77	<0.001

MACCE: in-hospital all-cause death, acute myocardial infarction or ischemic stroke.

*p<0.05 for difference when comparing COPD patients and non-COPD patients

Relative change overtime (%) is the difference from period 2011–15 to 2001–05 divided by the figure in period 2011/15

Patient age increased significantly in patients with and without COPD (means 65.84 years and 63.52 years, respectively, in 2001–05 vs. 67.26 and 65.48 years in 2011–15). Female sex decreased significantly in non-COPD patients, from 65.48% in 2001–05 to 61.71% in 2011–15.

We found a significant increase in the frequency of SAVR, other valves procedures on pulmonary or tricuspid valves, and pacemaker implantation in patients with and without COPD over time (Table 2).

Mean LOHS for patients with and without COPD undergoing mechanical mitral valve replacement was 24.87 and 22.88 days, respectively, in the period from 2001–05, decreasing to 19.79 and 20.42 days, respectively, in 2013–15 (all p<0.001). The overall mean LOHS was significantly higher in patients with COPD (23.05 vs. 22.02 days).

In COPD patients, IHM and MACCE after mechanical mitral valve replacement decreased significantly (all p<0.001) over time from 14.06% and 18.59%, respectively, in 2001–05 to 9.02% and 9.64% in 2011–15. Among patients without COPD, also IHM and MACCE decreased significantly over time. No differences were found between the two groups of patients (Table 2).

The most common associated comorbidities for hospitalized COPD patients who underwent mechanical mitral valve replacement were atrial fibrillation (57.14%), pulmonary hypertension (27.22%) and coronary artery disease (25.24%) (Table 3).

**Table 3:** Comorbidity of patients hospitalized that underwent a mechanical mitral valve replacement in Spain from 2001 to 2015 according to COPD status.

	COPD	TOTAL	Relative change over time (%)	Trend
Charlson Comorbidity Index, mean (SD)*	Yes	0.78(0.65)	15.71	<0.001
	No	0.93(0.83)	9.2	<0.001
Type 2 diabetes mellitus, n(%)*	Yes	479(19.82)	25.4	0.015
	No	5275(15.72)	28.12	<0.001
Peripheral vascular disease, n(%)*	Yes	134(5.54)	43.27	0.019
	No	1088(3.24)	47.07	<0.001
Acute renal disease, n(%)	Yes	251(10.38)	35.98	0.003
	No	3613(10.76)	53.03	<0.001
Cerebrovascular disease, n(%)	Yes	105(4.34)	-0.44	0.834
	No	1618(4.82)	17.43	0.001
Congestive heart failure, n(%)*	Yes	586(24.24)	24.16	0.007
	No	7145(21.29)	13.58	<0.001
Atrial fibrillation, n(%)	Yes	1381(57.14)	5.25	0.323
	No	19685(58.65)	3.62	<0.001
Pulmonary hypertension, n (%)	Yes	658(27.22)	7.9	0.318
	No	9367(27.91)	4.94	0.016
Coronary artery disease, n (%)*	Yes	610(25.24)	13.83	0.043
	No	6223(18.54)	10.78	<0.001
Obesity, n(%)*	Yes	224(9.27)	35.26	0.016
	No	2275(6.78)	42.15	<0.001
Cardiogenic shock, n(%)	Yes	80(3.31)	-84.95	0.061
	No	1327(3.95)	18.48	0.008
Gastrointestinal hemorrhage, n(%)*	Yes	17(0.7)	7.78	0.389
	No	126(0.38)	-57.14	0.123
Endocarditis, n(%)	Yes	269(11.13)	9.63	0.448
	No	3346(9.97)	44.86	<0.001
Pneumonia, n(%)*	Yes	87(3.6)	-99.59	0.029
	No	903(2.69)	-48.28	<0.001
Renal disease, n(%)*	Yes	220(9.1)	46.41	<0.001
	No	1979(5.9)	46.62	<0.001
Liver disease, n(%)	Yes	87(3.6)	41.67	0.001
	No	1184(3.53)	43.86	<0.001
Cancer, n(%)	Yes	26(1.08)	-23.38	0.347
	No	301(0.9)	38.32	0.003
Weight loss, n(%)	Yes	12(0.5)	25,00	0.743
	No	133(0.4)	18.18	0.665

*p<0.05 for difference when comparing COPD patients and non-COPD patients

Relative change overtime (%) is the difference from period 2011–15 to 2001–05 divided by the figure in period 2011/15

Overall patients with COPD had less coexisting medical conditions (mean CCI 0.78±0.65 vs. 0.93±0.83) (p<0.001) but had higher rates of higher rates of type 2 diabetes mellitus (19.82% vs. 15.72%; p<0.001), peripheral vascular disease (5.54% vs. 3.24%; p<0.001), congestive heart failure (24.24% vs. 21.29%; p = 0.001), coronary artery disease (25.24% vs. 18.54%; p<0.001), obesity (9.27% vs. 6.78%; p<0.001), gastrointestinal hemorrhage (0.7% vs. 0.38%; p = 0.013), pneumonia (3.6% vs. 2.69%; p = 0.008) and renal disease (9.1% vs. 5.9%, p<0.001) (Table 3).

We detected a significant increase in comorbidity according to the mean CCI over time in both patients with and without COPD (p<0.001). The frequency of type 2 diabetes mellitus, peripheral vascular disease, acute renal disease, congestive heart failure, coronary artery disease, obesity, renal disease and liver disease also increased significantly in both groups over time. In non-COPD patients we found a significant increase in frequency of cerebrovascular disease (from 4.5% in 2001–05 to 5.45% in 2011–15), atrial fibrillation (from 57.18% to 59.33%), pulmonary hypertension (from 26.94% to 28.34%), cardiogenic shock (from 3.53% to 4.33%), endocarditis (from 7.19% to 13.04) and cancer (from 0.66% to 1.07%) (Table 3).

The frequency of pneumonia decreased in both COPD and non-COPD patients from 4.89% and 3.01%, respectively, in 2001–05 to 2.45% and 2.03% in 2011–15 (Table 3).

### Trends in bioprosthetic mitral valve replacement hospitalizations

Bioprosthetic valves represented 9.78% of all valve replacements in period 2001–05 and increased to 22.39% in 2011–15 (p<0.001). Significant increments were observed for COPD patients from 11.41% to 25.82% and for non-COPD patients from 9.91% to 22.14% (p<0.001, in both cases).

S2 Fig shows a significant and constant increase in the hospitalization rates over time for COPD and non-COPD patients who underwent bioprosthetic mitral valve replacement (from 0.69 and 1.2 cases per 100,000 inhabitants in 2001 to 5.59 and 3.46 cases in 2015; p<0.001).

Compared to patients without COPD the incidence of bioprosthetic mitral valve replacement hospitalizations was lower among COPD patients (IRR 0.89;95%CI 0.82–0.98).

In patients who underwent bioprosthetic mitral valve replacement, there was a significant male predominance (76.82% for COPD and 38.27% for non-COPD patients). In non-COPD patients mean age increased significantly (p<0.001) from 72.12 years in 2001–05 to 74.42 years in 2011–15. We found a significant increase in the frequency of SAVR, other valves procedures on pulmonary or tricuspid valves and blood transfusion in patients without COPD patients over time. No differences were found among COPD patients. (Table 4).

**Table 4:** Incidence, sociodemographic and clinical characteristics of patients hospitalized that underwent a bioprosthetic mitral valve replacement in Spain from 2001 to 2015 according to COPD status.

	COPD	TOTAL	Relative change overtime (%)	Trend
Number of procedures (Incidence per 100,000 inhabitants) *	Yes	548(1.92)	15.71	<0.001
	No	6493(2.15)	9.2	<0.001
	Both	7041(2.70)	25.4	<0.001
Age, mean (SD)	Yes	73.59(5.75)	28.12	0.27
	No	73.62(7.07)	43.27	<0.001
Female sex, n (%)*	Yes	127(23.18)	47.07	0.246
	No	4008(61.73)	35.98	0.800
Coronary artery bypass graft, n(%)*	Yes	124(22.63)	53.03	0.644
	No	1170(18.02)	-0.44	0.987
Surgical aortic valve replacement, n(%)	Yes	181(33.03)	17.43	0.294
	No	2262(34.84)	24.16	0.002
Other valves procedures on pulmonary or tricuspid valves, n(%)*	Yes	117(21.35)	13.58	0.054
	No	1789(27.55)	5.25	<0.001
Intra-aortic balloon counterpulsation, n(%)	Yes	33(6.02)	3.62	0.948
	No	362(5.58)	7.9	0.051
Pacemaker implantation, n(%)	Yes	39(7.12)	4.94	0.505
	No	424(6.53)	13.83	0.279
Blood transfusion, n(%)	Yes	133(24.27)	10.78	0.746
	No	1746(26.89)	35.26	<0.001
Length of hospital stay, mean (SD)	Yes	25.64(23.87)	42.15	0.033
	No	24.39(23.28)	-84.95	<0.001
In-hospital mortality, n(%)*	Yes	98(17.88)	18.48	0.094
	No	957(14.74)	7.78	0.002
MACCE, n(%)	Yes	119(21.72)	-57.14	0.027
	No	1246(19.19)	9.63	<0.001

MACCE: in-hospital all-cause death, acute myocardial infarction or ischemic stroke.

*p<0.05 for difference when comparing COPD patients and non-COPD patients

Relative change overtime (%) is the difference from period 2011–15 to 2001–05 divided by the figure in period 2011/15

In patients with and without COPD, mean LOHS decreased significantly over time (30.95 and 26. 56 days, respectively, in the first period vs. 24.65 and 22.73 days in the last one) (Table 4).

For the total time period, crude IHM was significantly higher in COPD patients than in non-COPD patients (17.88% vs. 14.74%; p = 0.048). In non-COPD patients, IHM decreased over time, however no significant changes were found in COPD patients. MACCE was 21.74% for COPD patients and 19.19% for non-COPD individuals. MACCE decreased significantly in both groups over time (Table 4).

Atrial fibrillation (55.47%), coronary artery disease (34.12%) and congestive heart failure (30.66%) were the most common associated comorbidities for COPD patients who underwent bioprosthetic valve. Overall, COPD patients had less coexisting medical conditions (mean CCI 0.93±0.73 vs. 1.13±0.9) (p<0.001) and had higher rates of type 2 diabetes mellitus (21.72% vs. 18.13%; p = 0.037), peripheral vascular disease (6.75% vs. 4.68%; p = 0.030), coronary artery disease (34.12% vs. 28.15%; p = 0.003), pneumonia (5.29% vs. 3.62%, p = 0.048) and renal disease (13.87% vs. 9.55%;p = 0.001) than non-COPD patients (Table 4). However non-COPD patients had higher rates of pulmonary hypertension (29.11% vs. 24.27%; p = 0.016) (Table 5).

**Table 5:** Comorbidity of patients hospitalized that underwent a bioprosthetic mitral valve replacement in Spain from 2001 to 2015 according to COPD status.

	COPD	TOTAL	Relative change overtime (%)	Trend
Charlson Comorbidity Index, mean (SD)*	Yes	0.93(0.73)	-15.28	0.657
	No	1.13(0.9)	8.51	<0.001
Type 2 diabetes mellitus, n(%)*	Yes	119(21.72)	-29.8	0.373
	No	1177(18.13)	18.15	0.020
Peripheral vascular disease, n(%)*	Yes	37(6.75)	43.19	0.404
	No	304(4.68)	27.22	0.134
Acute renal disease, n(%)	Yes	113(20.62)	39.5	0.140
	No	1165(17.94)	37.92	<0.001
Cerebrovascular disease, n(%)	Yes	22(4.01)	16.67	0.878
	No	345(5.31)	24.56	0.171
Congestive heart failure, n(%)	Yes	168(30.66)	2.92	0.900
	No	1820(28.03)	14.9	0.004
Atrial fibrillation, n(%)	Yes	304(55.47)	18.28	0.155
	No	3671(56.54)	15.16	<0.001
Pulmonary hypertension, n (%)*	Yes	133(24.27)	34.05	0.175
	No	1890(29.11)	14.34	0.009
Coronary artery disease, n (%)*	Yes	187(34.12)	-12.07	0.651
	No	1828(28.15)	2.71	0.877
Obesity, n(%)	Yes	42(7.66)	66.7	0.011
	No	400(6.16)	31.51	0.006
Cardiogenic shock, n(%)	Yes	32(5.84)	-16.55	0.941
	No	341(5.25)	-14.72	0.568
Gastrointestinal hemorrhage, n(%)	Yes	7(1.28)	-66.67	0.837
	No	38(0.59)	-228.21	0.002
Endocarditis, n(%)	Yes	108(19.71)	31.05	0.336
	No	1230(18.94)	38.87	<0.001
Pneumonia, n(%)*	Yes	29(5.29)	-127.52	0.116
	No	235(3.62)	-25.28	0.232
Renal disease, n(%)*	Yes	76(13.87)	47.92	0.032
	No	620(9.55)	42.87	<0.001
Liver disease, n(%)	Yes	25(4.56)	16.73	0.441
	No	245(3.77)	22.95	0.135
Cancer, n(%)	Yes	5(0.91)	-150,00	0.512
	No	84(1.29)	26.15	0.449
Weight loss, n(%)	Yes	1(0.18)	100,00	0.597
	No	25(0.39)	47.83	0.574

*p<0.05 for difference when comparing COPD patients and non-COPD patients

Relative change overtime (%) is the difference from period 2011–15 to 2001–05 divided by the figure in period 2011/15

In patients with and without COPD the prevalence of obesity and renal disease increased significantly over time.

In non-COPD patients, the prevalence of type 2 diabetes mellitus, acute renal disease, congestive heart failure, atrial fibrillation, pulmonary hypertension and endocarditis also increased significantly over time. However the prevalence of gastrointestinal hemorrhage decreased from 1.28% in 2001–05 to 0.39% in 2011–15 (p = 0.002) in patients without COPD. No differences were found among COPD patients (Table 5).

### Differences between COPD patients admitted for mechanical versus bioprosthetic mitral valve replacement

When we compared COPD patients who underwent mechanical mitral valve replacement with men who underwent bioprosthetic mitral valve replacement, we found that the mechanical valve replacement patients were younger than patients who received bioprosthetic valves (66.5 years vs. 73.59 years; p<0.001). COPD patients who received bioprosthetic versus mechanical valves had more comorbidity (mean CCI, 0.93±0.73 vs. 0.78±0.0.65) and more likely require concomitant CABG (22.63% vs. 16.26%), intra-aortic balloon counterpulsation (6.02% vs. 3.39%) and pacemaker implantation (7.12% vs. 4.24%). In addition, they had higher IHM (17.88% vs. 11.54%) and higher values of MACCE (21.72% vs. 15.23%) compared with those men who received mechanical valves (Table 2 and Table 4).

### Factors associated with IHM

The factors independently associated with IHM according to the type of mitral valve are shown in Tables 6 and 7.

**Table 6:** Multivariable analysis of factors associated with IHM among COPD and non-COPD patients who underwent mechanical mitral valve replacement.

	COPD	No COPD	Both
	OR (95%CI)	OR (95%CI)	OR (95%CI)
Age	1.06(1.05–1.06)	1.06(1.04–1.08)	1.06(1.05–1.06)
Female sex	1.06(0.98–1.16)	1.07(0.79–1.46)	1.06(0.97–1.15)
Surgical aortic valve replacement	1.16(1.07–1.27)		1.16(1.08–1.27)
Other valves procedures on pulmonary or tricuspid valves	1.2(1.09–1.32)		1.2(1.1–1.31)
Intra-aortic balloon counterpulsation	2.94(2.52–3.44)	4.17(2.33–7.46)	3(2.58–3.49)
Pacemaker implantation	0.57(0.46–0.71)		0.59(0.48–0.73)
Blood transfusion	1.48(1.36–1.62)	1.91(1.42–2.58)	1.51(1.39–1.64)
Peripheral vascular disease	1.27(1.05–1.54)		1.23(1.03–1.48)
Acute Renal disease	3.93(3.57–4.32)	3.59(2.54–5.07)	3.9(3.56–4.28)
Cerebrovascular disease	1.69(1.45–1.98)		1.68(1.45–1.96)
Congestive heart failure	1.59(1.46–1.73)	1.43(1.05–1.94)	1.57(1.45–1.71)
Atrial fibrillation	0.49(0.46–0.54)	0.75(0.56–0.99)	0.51(0.47–0.55)
Coronary artery disease	1.39(1.27–1.53)		1.47(1.31–1.65)
Cardiogenic shock	6.29(5.47–7.23)	8.77(5.07–15.18)	6.4(5.6–7.33)
Gastrointestinal hemorrhage	2.86(1.89–4.34)		2.84(1.93–4.2)
Endocarditis	1.82(1.62–2.03)	1.99(1.37–2.9)	1.82(1.64–2.03)
Pneumonia	2.96(2.5–3.5)	2.39(1.39–4.13)	2.88(2.45–3.38)
Renal disease	1.76(1.54–2)		1.69(1.49–1.91)
Liver disease	4.04(3.47–4.72)	3.46(1.92–6.25)	4(3.45–4.65)
Cancer	1.46(1.04–2.05)		1.45(1.05–2.02)
Weight loss	1.63(1.05–2.54)		1.55(1.01–2.38)
Year	0.67(0.64–0.7)	0.68(0.57–0.82)	0.67(0.64–0.7)
COPD			0.99(0.86–1.16)

**Table 7:** Multivariable analysis of factors associated with IHM among COPD and non-COPD patients who underwent bioprosthetic mitral valve replacement.

	COPD	No COPD	Both
	OR (95%CI)	OR (95%CI)	OR (95%CI)
Age	1.02(1.01–1.04)	1.05(1.01–1.1)	1.02(1.01–1.04)
Female sex	1.38(1.17–1.64)	1.13(0.62–2.05)	1.37(1.16–1.61)
Surgical aortic valve replacement	1.3(1.1–1.53)		1.29(1.1–1.51)
Intra-aortic balloon counterpulsation	1.05(3.11–5.31)	3.52(1.44–8.62)	3.92(3.04–5.06)
Pacemaker implantation	0.46(0.31–0.69)	0.22(0.05–0.98)	0.43(0.3–0.64)
Blood transfusion	1.35(1.14–1.6)		1.34(1.14–1.57)
Peripheral vascular disease	1.67(1.2–2.32)		1.52(1.12–2.09)
Acute Renal disease	2.85(2.39–3.4)	3.14(1.83–5.38)	2.84(2.4–3.36)
Cerebrovascular disease	1.6(1.18–2.17)		1.62(1.21–2.17)
Congestive heart failure	1.74(1.48–2.05)		1.62(1.39–1.9)
Atrial fibrillation	0.59(0.5–0.7)		0.61(0.52–0.71)
Coronary artery disease	1.25(1.05–1.49)		1.22(1.04–1.45)
Cardiogenic shock	6.16(4.71–8.08)	4.19(1.68–10.45)	5.89(4.55–7.62)
Endocarditis	1.51(1.25–1.82)	1.8(1.03–3.15)	1.52(1.28–1.83)
Pneumonia	3.17(2.31–4.35)		2.98(2.22–4.03)
Renal disease	1.54(1.23–1.96)		1.57(1.26–1.96)
Liver disease	3.5(2.55–4.82)	3.93(1.5–10.3)	3.53(2.61–4.77)
Year	0.71(0.64–0.78)	0.63(0.47–0.86)	0.7(0.63–0.77)
COPD			1.32(1.01–1.73)

Regardless of the type of valve implanted and for both patients with and without COPD, factors that increased IHM included older age, intra-aortic balloon counterpulsation during admission, acute renal disease, cardiogenic shock, endocarditis and liver disease.

Blood transfusion during admission, congestive heart failure and pneumonia increase the risk of IHM in patients with and without COPD with mechanical mitral valve replacement. Among COPD patients, IHM was significantly higher in those who underwent SAVR (OR 1.16;95%CI 1.07–1.27), who underwent other valves procedures on pulmonary or tricuspid valves (OR 1.2;95%CI 1.09–1.32), with peripheral vascular disease (OR 1.27;95%CI 1.05–1.54), with cerebrovascular disease (OR 1.69;95%CI 1.45–1.98), with coronary artery disease (OR 1.39;95%CI 1.27–1.53), with gastrointestinal hemorrhage (OR 2.86;95%CI 1.89–4.34), with renal disease (OR 1.76;95%CI 1.54–2), cancer (OR 1.46; 95%CI 1.04–2.05) and weight loss (OR 1.63;95%CI 1.05–2.54).

Among COPD patients who underwent bioprosthetic valve, IHM was significantly higher in female sex, those who underwent SAVR, with blood transfusion during admission, with peripheral vascular disease, with cerebrovascular disease, with congestive heart failure, with coronary artery disease, with pneumonia and with renal disease.

Atrial fibrillation was associated with a lower risk of dying for both COPD patients and non-COPD patients who underwent mechanical valve replacement (OR 0.49;95%CI 0.46–0.54 and OR 0.75;95%CI 0.56–0.99, respectively). Among COPD patients underwent mechanical valve replacement pacemaker implantation was associated with lower IHM (OR 0.57;95%CI 0.46–0.71).

Pacemaker implantation was associated with a lower risk of IHM in patients with and without COPD with bioprosthetic mitral valve replacement (OR 0.46; 95%CI 0.31–0.69 and OR 0.22;95%CI 0.05–0.98, respectively). Furthermore, atrial fibrillation was also associated with a lower risk of dying in patients with COPD (OR 0.59; 95%CI 0.5–0.7).

Over time, the IHM decreased significantly regardless of the type of valve.

After multivariable adjustment, COPD was associated with a significantly higher IHM (OR 1.32;95%CI 1.01–1.73) among patients who underwent bioprosthetic mitral valve replacement in our study. However, COPD did not predict IHM among those patients who underwent mechanical mitral valve replacement (OR 0.99; 95%CI 0.86–1.16).

Finally, the results of the sensitivity analysis using PSM (S1 Table) confirmed the results of the multivariable logistic regression with significantly higher IHM among COPD patients who received a bioprosthetic valve than among non-COPD patients.

## Discussion

In this large analysis of patients undergoing SMVR, we demonstrated that COPD was associated with a significantly higher IHM among patients who received a bioprosthetic valve, but this disease did not predict IHM among those receiving a mechanical valve. These results contrast with those obtained by other authors who have not found that COPD is a predictor of mortality in patients undergoing SMVR with biological or mechanical substitutes [17]. Goldstone et al [5] also found that mortality was lower among patients up to 70 years of age who received a mechanical mitral valve than among those who received a biologic prosthesis, although they did not stratify the results by the existence or not of COPD.

Compared to patients without COPD, the incidence of mitral valve replacement hospitalizations was lower among COPD patients in our study, regardless the type of valve used. The presence of comorbidities, as attested by a higher CCI, has been associated with a more frequent decision not to operate in patients with severe symptomatic mitral regurgitation [9]. This may because an increased CCI has a negative impact on survival [14] and most comorbidities may also increase operative risk [18], including chronic airway disease [19].

Despite the limited durability of biological valves, increasing numbers of younger patients are choosing to receive them, due mainly to the lack of a need for permanent anticoagulation [20]. In this way, we observed an increase of the incidence of bioprosthetic mitral valve replacement among COPD and non-COPD patients from 2001 through 2015. By contrast, the incidence of mechanical mitral valve replacement decreased in both groups of patients during the study period. Goldstone et al [5] also reported that the use of biologic prostheses increased substantially for mitral-valve replacement from 1996 through 2013 in California. Justification for this approach may be related to the improved durability of the current generation of tissue valves and the technical feasibility of a subsequent valve-to-valve procedure, in the event that occurs a degeneration of the tissue valve, to avoid having to resort an open-heart surgery [7]. Although this approach may be reasonable in high-risk and elderly patients, as often it occurs in COPD patients, it is not supported by scientific evidence in younger patients, especially in those without comorbidities or contraindications for anticoagulation [7]. In any case, the choice of replacement mitral valve type is a decision that should be taken by physician and patient in conjunction and must be individualized. For this, it should be accounting the risks of reoperation and of chronic anticoagulation and their consequences, in addition to characteristics of the patient, lifestyles, comorbidities and life expectancy, in order to increase quality of life and survival [17].

In our study, COPD patients underwent CABG more frequently than those without COPD, both for mechanical and bioprosthetic valves. However, they underwent other valves procedures on pulmonary or tricuspid valves less frequently than non-COPD patients. In this way, Sá et al [21] found that mitral valve replacement may be performed safely, concomitantly with CABG, in patients with moderate-to-severe ischemic mitral regurgitation. In such patients, the combined procedure resulted in lower rates of postoperative atrial fibrillation and low cardiac output than CABG only. In addition, although both surgical approaches resulted in significant improvement in postoperative left ventricular ejection fraction, there was greater improvement in the combined surgery group, Thereby, despite being a more aggressive approach, the combined surgical procedure did not increase morbidity or mortality.

IHM following mitral valve replacement is high [12]. We found a significant decrease in IHM over time among COPD and non-COPD patients who underwent a mechanical or bioprosthetic mitral valve replacement. Berzingi et al [12] also demonstrated a significant decrease in IHM between 2003–2014 for isolated bioprosthetic mitral valve replacement. On the other hand, as it has been described in previous studies [22], being older and having additional concomitant procedures, were associated with a higher mortality risk in ours. Furthermore, several comorbidities were found to be associated with increased mortality risk in our multivariable analysis. These data might be useful when interpreting the emerging literature of transcatheter mitral valve replacement [12].

### Limitations

The findings of this study must be viewed in light of limitations inherent to retrospective observational studies that rely on administrative data. We used the SNHDD, an administrative database rather than a clinical database. Therefore, it was subject to coding error. Our definition of COPD was not based on lung function test because SNHDD does not include the results of diagnostic tests. Therefore, clinical measures of severity and type of COPD were not available. We also did not have data on COPD treatment duration and medication dosing requirements, (e.g., dose and duration of steroid use). Implied in this information deficit is the inability to determine whether COPD symptoms were caused by chronic heart (CHF) failure and distinguishing symptoms caused by CHF but attributed to COPD.

However, we have followed similar designs of other descriptive studies on COPD hospitalizations [23–28]. Additionally, in several studies it has been assessed the validity of healthcare databases for COPD [29–34]. In this way, administrative databases seem beneficial for planning health care interventions, including the COPD field. They constitute robust information systems subjected to periodic data quality controls [13] remaining the prevalent data source, reliable because of the amount of data and the population coverage, especially in countries with a National Health System [35], like ours. Thereby, this dataset reflects the outcome of the world-real population, which is different from that selected in randomized controlled trials. Thus, the results could be extrapolated to the general population.

### Conclusions

In conclusion, the incidence of mechanical SMVR decreased while that of bioprosthetic SMVR increased in COPD and non-COPD patients from 2001 to 2015 in Spain. Patients with COPD were less surgically operated than those without COPD for both valve types. In COPD patients, bioprosthetic SMVR was proportionally more used than mechanical SMVR. Mortality decreased over time for both valve types in patients with and without COPD. COPD was associated with IHM in patients undergoing a biological SMVR, but not in those that underwent a mechanical SMVR.

## Supporting information

S1 Fig Incidence of mechanical mitral valve replacement among COPD and non-COPD patients in Spain 2001–2015. (DOCX)

S2 Fig Incidence of bioprosthetic mitral valve replacement among COPD and non-COPD patients in Spain 2001–2015. (DOCX)

S1 Table Distribution according to study variables of propensity score–matched COPD and non-COPD patients who underwent a mechanical or bioprosthetic surgical mitral valve replacement. (DOCX)

## Abbreviations

CABG: Coronary Artery Bypass Graft
CCI: Charlson comorbidity index
COPD: chronic obstructive pulmonary disease
ICD-9-CM: International Classification of Diseases, Ninth Revision, Clinical Modification
IHM: in-hospital mortality
LOHS: length of hospital stay
MACCE: Major Adverse Cardiovascular and Cerebrovascular Events
SAVR: Surgical Aortic Valve Replacement
SMVR: surgical mitral valve replacement
SNHDD: Spanish National Hospital Discharge Database
