# Supplementary material for: Trends, characteristics, in-hospital outcomes and mortality in surgical mitral valve replacement among patients with and without COPD in Spain (2001-2015)
Source: PLoS One. 2019 Aug 19;14(8):e0221263. doi: 10.1371/journal.pone.0221263 (PMC6699799; doi:10.1371/journal.pone.0221263)
Supplement: S2 Fig — (DOCX) [file pone.0221263.s002.docx]

Figure 2 in S2 Figure. Incidence of bioprosthetic mitral valve replacement among COPD and non-COPD patients in Spain 2001-2015
