# Supplementary material for: Trends, characteristics, in-hospital outcomes and mortality in surgical mitral valve replacement among patients with and without COPD in Spain (2001-2015)
Source: PLoS One. 2019 Aug 19;14(8):e0221263. doi: 10.1371/journal.pone.0221263 (PMC6699799; doi:10.1371/journal.pone.0221263)
Supplement: S1 Table — (DOCX) [file pone.0221263.s003.docx]

Supplementary Table 1. Distribution according to study variables of propensity score–matched COPD and non-COPD patients who underwent a mechanical or bioprosthetic surgical mitral valve replacement.

|  |  | Mechanical | |  | Bioprosthetic | |  |
| --- | --- | --- | --- | --- | --- | --- | --- |
|  |  | COPD | NON-COPD | P | COPD | NON-COPD | P |
| Age, , mean (SD) |  | 66.5(8.43) | 66.62(8.68) | 0.602 | 73.59(5.75) | 73.86(7.13) | 0.490 |
| Sex, n(%) | Male | 1629(67.4) | 1641(67.89) | 0.712 | 421(76.82) | 419(76.46) | 0.887 |
|  | Female | 788(32.6) | 776(32.11) |  | 127(23.18) | 129(23.54) |  |
| Coronary artery bypass graft, n(%) | No | 2024(83.74) | 2025(83.78) | 0.969 | 424(77.37) | 440(80.29) | 0.237 |
|  | Yes | 393(16.26) | 392(16.22) |  | 124(22.63) | 108(19.71) |  |
| Surgical aortic valve replacement, n(%) | No | 1639(67.81) | 1665(68.89) | 0.421 | 367(66.97) | 354(64.6) | 0.408 |
|  | Yes | 778(32.19) | 752(31.11) |  | 181(33.03) | 194(35.4) |  |
| Other valves procedures on pulmonary or tricuspid valves, n(%) | No | 2230(92.26) | 2212(91.52) | 0.343 | 509(92.88) | 500(91.24) | 0.315 |
|  | Yes | 187(7.74) | 205(8.48) |  | 39(7.12) | 48(8.76) |  |
| Intra-aortic ballon counterpulsation, n(%) | No | 2335(96.61) | 2339(96.77) | 0.748 | 515(93.98) | 508(92.7) | 0.397 |
|  | Yes | 82(3.39) | 78(3.23) |  | 33(6.02) | 40(7.3) |  |
| Pacemaker implantation, n(%) | No | 2303(95.28) | 2303(95.28) | 0.999 | 509(92.88) | 512(93.43) | 0.720 |
|  | Yes | 114(4.72) | 114(4.72) |  | 39(7.12) | 36(6.57) |  |
| Charlson Comorbidity Index, mean (SD) |  | 1.78(0.85) | 1(0.95) | 0.000 | 1.93(0.93) | 1.22(1.04) | 0.000 |
| Type 2 diabetes mellitus, n (%) | No | 1938(80.18) | 1981(81.96) | 0.114 | 429(78.28) | 436(79.56) | 0.605 |
|  | Yes | 479(19.82) | 436(18.04) |  | 119(21.72) | 112(20.44) |  |
| Peripheral vascular disease, n(%) | No | 2283(94.46) | 2300(95.16) | 0.271 | 511(93.25) | 504(91.97) | 0.419 |
|  | Yes | 134(5.54) | 117(4.84) |  | 37(6.75) | 44(8.03) |  |
| Cerebrovascular disease, n(%) | No | 2312(95.66) | 2313(95.7) | 0.944 | 526(95.99) | 528(96.35) | 0.753 |
|  | Yes | 105(4.34) | 104(4.3) |  | 22(4.01) | 20(3.65) |  |
| Congestive heart failure, n(%) | No | 1831(75.76) | 1852(76.62) | 0.478 | 380(69.34) | 372(67.88) | 0.603 |
|  | Yes | 586(24.24) | 565(23.38) |  | 168(30.66) | 176(32.12) |  |
| Atrial fibrillation, n(%) | No | 1036(42.86) | 1027(42.49) | 0.794 | 244(44.53) | 250(45.62) | 0.716 |
|  | Yes | 1381(57.14) | 1390(57.51) |  | 304(55.47) | 298(54.38) |  |
| Pulmonary hypertension, n (%) | No | 1759(72.78) | 1795(74.27) | 0.241 | 415(75.73) | 417(76.09) | 0.888 |
|  | Yes | 658(27.22) | 622(25.73) |  | 133(24.27) | 131(23.91) |  |
| Coronary artery disease, n (%) | No | 1807(74.76) | 1797(74.35) | 0.741 | 361(65.88) | 369(67.34) | 0.609 |
|  | Yes | 610(25.24) | 620(25.65) |  | 187(34.12) | 179(32.66) |  |
| Obesity, n(%) | No | 2193(90.73) | 2216(91.68) | 0.243 | 506(92.34) | 504(91.97) | 0.822 |
|  | Yes | 224(9.27) | 201(8.32) |  | 42(7.66) | 44(8.03) |  |
| Cardiogenic shock, n(%) | No | 2337(96.69) | 2351(97.27) | 0.239 | 516(94.16) | 514(93.8) | 0.800 |
|  | Yes | 80(3.31) | 66(2.73) |  | 32(5.84) | 34(6.2) |  |
| Endocarditis, n(%) | No | 2148(88.87) | 2187(90.48) | 0.065 | 440(80.29) | 441(80.47) | 0.939 |
|  | Yes | 269(11.13) | 230(9.52) |  | 108(19.71) | 107(19.53) |  |
| Pneumonia, n(%) | No | 2330(96.4) | 2331(96.44) | 0.938 | 519(94.71) | 512(93.43) | 0.371 |
|  | Yes | 87(3.6) | 86(3.56) |  | 29(5.29) | 36(6.57) |  |
| Renal disease, n(%) | No | 2197(90.9) | 2224(92.01) | 0.168 | 472(86.13) | 466(85.04) | 0.666 |
|  | Yes | 220(9.1) | 193(7.99) |  | 76(13.87) | 82(14.96) |  |
| Liver disease, n(%) | No | 2330(96.4) | 2334(96.57) | 0.755 | 523(95.44) | 530(96.72) | 0.277 |
|  | Yes | 87(3.6) | 83(3.43) |  | 25(4.56) | 18(3.28) |  |
| Cancer, n(%) | No | 2391(98.92) | 2394(99.05) | 0.667 | 543(99.09) | 546(99.64) | 0.256 |
|  | Yes | 26(1.08) | 23(0.95) |  | 5(0.91) | 2(0.36) |  |
| Length of hospital stay, mean (SD) |  | 23.05(19.72) | 22.94(22.38) | 0.851 | 25.64(23.87) | 26.29(24.44) | 0.653 |
| In-hospital mortality, n(%) | No | 2138(88.46) | 2127(88) | 0.868 | 450(82.12) | 483(88.14) | 0.006 |
|  | Yes | 279(11.54) | 290(12) |  | 98(17.88) | 65(11.86) |  |
| MACCE, n(%) | No | 2049(84.77) | 2029(83.95) | 0.428 | 429(78.28) | 437(79.74) | 0.553 |
|  | Yes | 368(15.23) | 388(16.05) |  | 119(21.72) | 111(20.26) |  |
